# Pulmonary Langerhans Cell Histiocytosis: An Unusual Differential for a Solitary Lung Nodule

**DOI:** 10.1155/2024/1315222

**Published:** 2024-01-27

**Authors:** Tessabella M. Magliochetti Cammarata, Sushan Gupta, Sai Sri Harsha Rallabhandi, Vishesh Paul

**Affiliations:** ^1^Carle Illinois College of Medicine, University of Illinois Urbana-Champaign, 506 S Mathews Ave, Urbana, IL 61801, USA; ^2^Department of Internal Medicine, Carle Foundation Hospital, 611 W Park St, Urbana, IL 61801, USA; ^3^Department of Pulmonary and Critical Care Medicine, Carle Foundation Hospital, 611 W Park St, Urbana, IL 61801, USA

## Abstract

**Background:**

Pulmonary Langerhans cell histiocytosis (LCH) commonly presents as bilateral reticulonodular and cystic lung changes on chest imaging. Isolated lung nodule presentation is rare. *Case Presentation*. Our patient was an elderly male and an active smoker, who was referred to the pulmonology clinic for an incidental 19 mm lung nodule seen on a chest CT scan. A CT-guided transthoracic needle biopsy was performed to rule out malignancy. The biopsy sample showed marked inflammatory infiltrate with abundant eosinophils and epithelioid histiocyte-like cells suggestive of Langerhans cell histiocytosis. Antibodies against CD1a and Langerhans were positive which confirmed the diagnosis. During follow-ups, the patient had reduced smoking, and the lung nodule had decreased in size to 14 mm.

**Conclusion:**

An isolated lung nodule in a patient with a smoking history always warrants a malignancy workup. Characteristic pathological findings with immunostaining are necessary to differentiate pulmonary LCH in these cases. Failure to perform immunostaining in such cases may lead to missing this vital diagnosis.

## 1. Introduction

Langerhans cell histiocytosis (LCH) is a rare neoplastic proliferation disorder of myeloid precursor cells [1]. LCH primarily affects children and has a systemic presentation. Isolated pulmonary involvement is seen mainly in adults, with a predilection for younger adults [1, 2]. Pulmonary LCH commonly presents as bilateral reticulonodular and cystic lung changes seen on a chest computerized tomography (CT) scan, especially in patients with a substantial smoking history. A solitary nodule due to pulmonary LCH is rare [2]. In such patients, lung malignancy is often the leading differential. We report an unusual case of pulmonary LCH presenting as an isolated lung nodule in an elderly individual with a significant smoking history to demonstrate the importance of performing immunostaining even with a unilateral presentation.

## 2. Case Presentation

**Figure 1:**
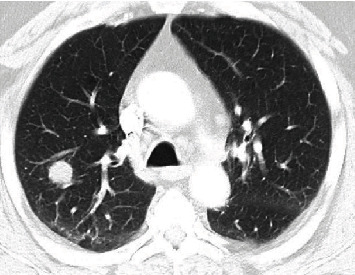
A large 19 mm nodule in the right upper lobe lung on noncontrast CT chest.

Our patient was an elderly male in his 60s who was referred to the pulmonology clinic for an incidental lung nodule seen on a chest CT scan. The patient had an emergency room visit for shortness of breath and cough two weeks before the pulmonology clinic visit. The CT chest scan performed at that time showed no acute infiltrate or pulmonary embolism but revealed emphysema and a right upper lobe lung nodule (Figure 1). The nodule was solid, noncalcified, and measured 19 mm in diameter. After treatment with systemic steroids and albuterol nebulization, he was discharged home with instructions to follow up in the pulmonology clinic.

At the pulmonology clinic visit, he denied any acute respiratory or constitutional symptoms. His only symptom was mild dyspnea on exertion, which had been going on for over a year. He denied any significant past medical history except latent tuberculosis, for which he had completed treatment many years ago. He was an active cigarette smoker with more than 40 pack-years of smoking history. He had worked as a mechanic for most of his adult life. His family history was significant for pancreatic and liver cancer in his mother and gastric cancer in his father. The patient had no previous CT scans for comparison, and the chest X-ray from a few years ago showed no apparent nodule.

On the exam, his vital signs were stable, with a SpO2 of 96% on room air. The physical examination was unremarkable, including normal bilateral breath sounds. Spirometry testing showed no obstruction or restriction with a normal DLCO. Recent laboratory testing during the ER visit did not reveal any abnormalities. Given his significant smoking history and absence of infectious symptoms, malignancy was high on the differential diagnosis list.

**Figure 2:**
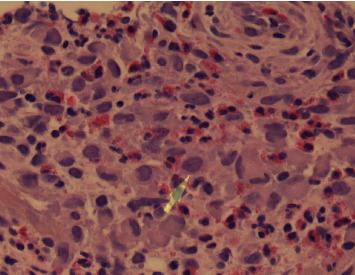
Eosinophils and epithelioid histiocyte-like cell infiltration mixed with fragments of blood clots (arrow-cleaved Langerhans cell nuclei).

**Figure 3:**
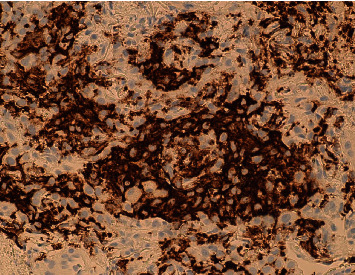
Diffuse immune reactivity against CD1a and Langerhans cells.

The calculated lung cancer risk based on the Brock University cancer prediction equation was 29.6%. Based on this high risk of malignancy, a CT-guided transthoracic needle biopsy was performed. Pathology showed marked inflammatory infiltrate with abundant eosinophils and epithelioid histiocyte-like cells suggestive of Langerhans cell histiocytosis (Figure 2). In addition, antibodies against CD1a and Langerhans showed diffuse reactivity within histiocytic infiltrate, which confirmed the diagnosis of pulmonary LCH (Figure 3). All other stains were negative. Mutation testing for MAPK genes, such as BRAF V600E, was also performed and was negative.

At the follow-up clinic appointment, we strongly advised complete smoking cessation, and he was able to cut down on smoking to 2-3 cigarettes a day over 6 months without medication support. He, however, was unable to quit altogether. A repeat chest CT scan completed after three months showed a reduction in the size of the nodule to 16 mm, and it further reduced to 14 mm on the scan completed one year later. The patient is now on annual surveillance with a low-dose CT scan and has no significant symptoms.

## 3. Discussion

Pulmonary LCH is a rare disorder characterized by imaging findings of irregularly shaped lung cysts or cystic nodules that classically present bilaterally and spare the lung bases and costophrenic angles [1, 2]. Emphysema and bronchial wall thickening are also commonly seen on imaging, as the disease is universally associated with smoking. Patients may be asymptomatic or present with mild dyspnea on exertion, chronic cough, or spontaneous pneumothorax. Pulmonary function testing often shows hyperinflation with no or minimal airway obstruction. Classic imaging findings in a patient with a significant smoking history are usually sufficient for diagnosis. However, transbronchial or surgical lung biopsies are often performed for confirmation.

Pathology in pulmonary LCH shows variable-sized stellate nodules (starfish-like), Langerhans cells, and acute and chronic inflammation. Langerhans cells are large cells with grooved nuclei and eosinophilic cytoplasm and stain positive for CD1a, langerin (CD 207), and S100 [1]. As the disease progresses, cysts, interstitial scarring, and emphysematous changes become more predominant, and Langerhans cells may not even be detectable. A subset of patients show mutations in the mitogen-activated protein kinase (MAPK) BRAF V600 E mutation, and among those, BRAF V600E and MAPK2K1 gene mutations are the most common.

Pulmonary LCH presenting as an isolated lung nodule is extremely rare, with only a handful of cases published in the literature [3–8]. In a patient with a substantial smoking history with a lung nodule, malignancy is the primary differential warranting a biopsy, as was done in our patient. The role of PET scans may be limited in differentiating LCH nodules from malignant lung nodules, as often both might be metabolically active, especially in the early diffuse nodular stage of pulmonary LCH [1]. In rare cases, PET scans can be negative in both malignancy and LCH, and a biopsy is often needed to confirm the diagnosis [6]. Additionally, PET scan can also help to identify easier access sites for biopsy; however, in our patient, after discussing with the patient the pros and cons of biopsy, and per patient preference, the decision was taken to pursue biopsy prior to performing PET scan.

Smoking cessation is the mainstay of therapy. Most patients will have disease stability or even resolution with smoking abstinence; however, there is no absolute correlation. In some patients, the disease may continue to progress despite smoking abstinence [1]. In progressive cases, corticosteroids and chemotherapy agents like cyclophosphamide, cladribine, cytarabine, and vinblastine have been tried with variable success. Lung transplant is reserved only for very severe cases [2]. Recently, targeted therapy has shown benefits in patients with identifiable genetic mutations like those mentioned above.

Our case adds to the scarce literature on this unusual and rare presentation of pulmonary LCH as a solitary lung nodule. Given the strong association with cigarette smoking, clinicians should still pursue to rule out malignancy in such individuals based on risk stratification. However, bedside clinicians and pathologists should remain cognizant of the rare solitary nodule presentation of LCH and confirm the diagnosis with immunostaining as needed.

## Data Availability

No datasets were used. General published literature data used to support the findings of this study are included within the article in the reference section.
